# Traditional games resulted in post-exercise hypotension and a lower cardiovascular response to the cold pressor test in healthy children

**DOI:** 10.3389/fphys.2014.00235

**Published:** 2014-06-25

**Authors:** Suliane B. Rauber, Daniel A. Boullosa, Ferdinando O. Carvalho, José F. V. N. de Moraes, Ioranny R. C. de Sousa, Herbert G. Simões, Carmen S. G. Campbell

**Affiliations:** ^1^Graduate Program on Physical Education and Health, Laboratory Study of Physical Activity and Health, Catholic University of BrasiliaBrasilia, Brazil; ^2^Undergraduate Program on Physical Education, Study Group of the Benefits of Physical Activity for Children's Health (GEBEXFISI-Grupo de Estudos dos Benefícios do Exercício Físico para Saúde Infantil), Catholic University of BrasiliaBrasilia, Brazil; ^3^Department of Physical Education, College of Physical Education, Federal University of Vale do São FranciscoPetrolina, Brazil

**Keywords:** cardiovascular system, blood pressure, heart rate, children, active playing

## Abstract

The present study aimed to verify if blood pressure (BP) reactivity could be reduced through a previous single session of active playing when compared to sedentary leisure. Sixteen pre-pubertal healthy children participated in this study. After familiarization with procedures and anthropometric evaluation, participants performed three sessions in randomized order: (1) 30 min of traditional Brazilian games (PLAY); (2) 30 min of video game playing (DDR); and (3) 30 min of watching TV (TV). Each session lasted 80 min, being 10 min of rest; 30 min of intervention activity; and 40 min of recovery. After recovery, the Cold Pressor Test (CPT) was used for the assessment of acute cardiovascular reactivity. BP was recorded at 30 s and 1 min during the CPT. Analysis of variance showed post-exercise hypotension (PEH) only after PLAY, and that systolic and diastolic BP were significantly increased in all conditions during CPT. However, the magnitude of the CPT-induced BP response was significantly less in PLAY compared to DDR and TV. The PEH observed during recovery and the reduced BP response to CPT following playing traditional games may be due its higher cardiovascular and metabolic demand as was indicated by the increased heart rate, oxygen consumption, and BP. It was concluded that BP reactivity to stress may be reduced through a previous single session of traditional games and that PEH was recorded only after this exercise form. This benefit indicates a potential role of playing strategies for cardiovascular health in childhood.

## Introduction

Cardiovascular disease (CVD) is the primary cause of death in western countries (Ergin et al., 2004). More recently, researchers have provided evidence that CVD is becoming more frequent among pediatric populations (Bigi et al., 2011) and the increase of blood pressure (BP) during infancy and puberty is becoming more premature (Kark et al., 2009; Maximova et al., 2009), and has been identified as the first etiologic factor for the development of hypertension in adulthood (Chiolero et al., 2008; Tsioufis et al., 2011). Therefore, it is important to identify factors that could influence cardiovascular health during childhood, specifically those lifestyle-related that are potentially modifiable (Bell and Belsky, 2008; Daniels, 2011; Moraes et al., 2014).

Physical inactivity is considered to be one of the main causes of primary hypertension, independently of weight status (Martinez-Gomez et al., 2009; Daniels, 2011). Physical activity (PA) levels are becoming lower among children and adolescents worldwide (Rossow et al., 2010; Nettle and Sprogis, 2011), demonstrating that new generations are more sedentary than the previous ones (Chinapaw et al., 2011). This raises concerns about which strategies could help children and adolescents to increase their PA levels, as PA in childhood has been demonstrated to be an effective intervention to combat obesity and hypertension in adult life (Guy et al., 2011; Siegrist et al., 2013).

Among several mechanisms related to the effectiveness of PA on BP control, there is the so-called post-exercise hypotension (PEH) (MacDonald, 2002). The effect of a single exercise bout induced BP reduction in relation to a non-exercise control session has been systematically observed after different exercise modes can last several hours post-exercise (Ciolac et al., 2008; Mota et al., 2009; Rabelo et al., 2009; Morais et al., 2011), thus helping for control BP in both hypertensive (Mota et al., 2009) and healthy people (Richter et al., 2010). However, the occurrence of PEH after exercise in children was not investigated yet to our knowledge.

An important application of PEH is that, during its occurrence, individuals are supposed to present lowered BP reactivity to different stress situations (MacDonald et al., 2001; Brownley et al., 2003). Stress can be considered an important factor that typically promotes a rise in BP (Lehman et al., 2009), which is dependent on the nature of the stimulus and the individual characteristics of the subject (Probst et al., 1997). Previous studies have demonstrated that an exercise bout of moderate intensity could attenuate the increase of BP and HR after a stressful event (Probst et al., 1997; Bond et al., 2002) in adults. More recently, the studies of Roemmich et al. (2009) and Lambiase et al. (2010) have showed a dampened cardiovascular reactivity to speech and stroop tasks after interval cycling and a self-paced walk respectively, in children. These important studies (Roemmich et al., 2009; Lambiase et al., 2010) highlighted the positive impact of different forms of exercise performed in laboratory conditions on acute stress reactivity. However, the short recovery interval used (20 min) in these previous studies did not allow a precise evaluation of the PEH phenomenon. Moreover, it would be interesting to evaluate also the impact of other activities more enjoyable to children, such as street games and active video game playing, that have been recently proposed to increase energy expenditure (Lanningham-Foster et al., 2006; Wang and Perry, 2006; Warburton et al., 2009; Rauber et al., 2013). This information would help to know the impact of other forms of exercise on both PEH and stress reactivity in children; and therefore to better understand the role of different exercise strategies for cardiovascular health during childhood.

On the other hand, the cold pressure test (CPT) is a simple and time-saving protocol proposed for the evaluation of cardiovascular reactivity to acute thermal stress (Hines and Brown, 1936; Wood et al., 1984) that requires passive coping in contrast to the active coping required in previous studies with children (Roemmich et al., 2009; Lambiase et al., 2010). Briefly, this test consists of the immersion of the left hand in cold water for the assessment of the BP responses (Hines and Brown, 1936). The validity of this test has been previously demonstrated as it is a good predictor of hypertension in adulthood from the recorded values during childhood (Wood et al., 1984; Menkes et al., 1989).

Thus, the purpose of present study was to analyze the occurrence of PEH after different games (i.e., video games and traditional Brazilian children's games), and to assess the potential protective effect of these games on cardiovascular reactivity to subsequent stress in children. The hypotheses were that both games would induce PEH and that cardiovascular reactivity would be lower after active games in comparison to the sedentary activity (TV).

## Methods

### Sample and ethic procedures

Sixteen children aged 9–10 years (8 boys and 8 girls) participated in this study. After authorization from the school, the children's parents received an informed consent letter describing all procedures, risks and benefits of the study and signed the consent for children's participation.

The exclusion criteria were: any physical impairment that could prevent the participant from performing the programmed activities; having any previous cardiometabolic disease (e.g., diabetes, hypertension); and any previous experience with the video game “Dance Dance Revolution” (DDR). The study was approved by the local ethics committee for human research (protocol 147/2009).

### General procedures

Four visits were necessary in order to perform the present study. The first one was for familiarization with the experimental procedures and for anthropometric evaluation. The other three visits were randomly performed between 8 and 11 a.m. Each visit lasted 80 min, divided in: rest (10 min); programmed activity (30 min); and recovery (40 min). Immediately after the 40th min of the post-activity recovery period, the CPT was applied over 1 min.

#### Familiarization and anthropometric evaluation

Evaluation of body weight (Electronic Scale Tech 05^®^, China), height (Portable Stadiometer, Sanny^®^, Brazil), and subscapular and triceps skinfolds (Adipometer, Lange Skinfold Caliper, USA) were performed for body composition estimation. Body fat percentage was estimated using the skinfolds method according to Slaughter et al. (1988). The CPT and BP measurements were also performed so the children were familiarized with these procedures. Subsequently, the children received orientations and practiced the DDR video game 10–15 times.

#### Traditional games (PLAY)

Volunteers participated of a physical education class together with other 10 children so the activity could be the most realistic possible. A physical education teacher guided an activity composed by three traditional games usually applied in physical education classes in Brazil, and organized as follows: 10 min of “run and catch;” 10 min of “dodge ball;” and 10 min of “capture the flag.” These activities were performed in the same order in a gymnasium close to university's laboratory. The intensity of the games was monitored by HR (Polar Electro Oy FS1, Finland), as well as through the responses of oxygen uptake (VO_2_), and respiratory exchange ratio (RER) (Cortex Metamax 3B, Germany).

#### “Dance Dance Revolution” video game (DDR)

Volunteers played an interactive video game (DDR) installed in a laptop computer. They selected the songs from a low-level difficulty list. Authors decided to use a basic level for standardization purposes, since their first contact with this video game was during the familiarization session.

#### Watching television (TV)

Volunteers watched two popular cartoons (“Sponge Bob” and “Ben 10”) for 30 min (15 min per cartoon) while seated in a quiet room.

#### BP and HR measurements

**Figure 1:**
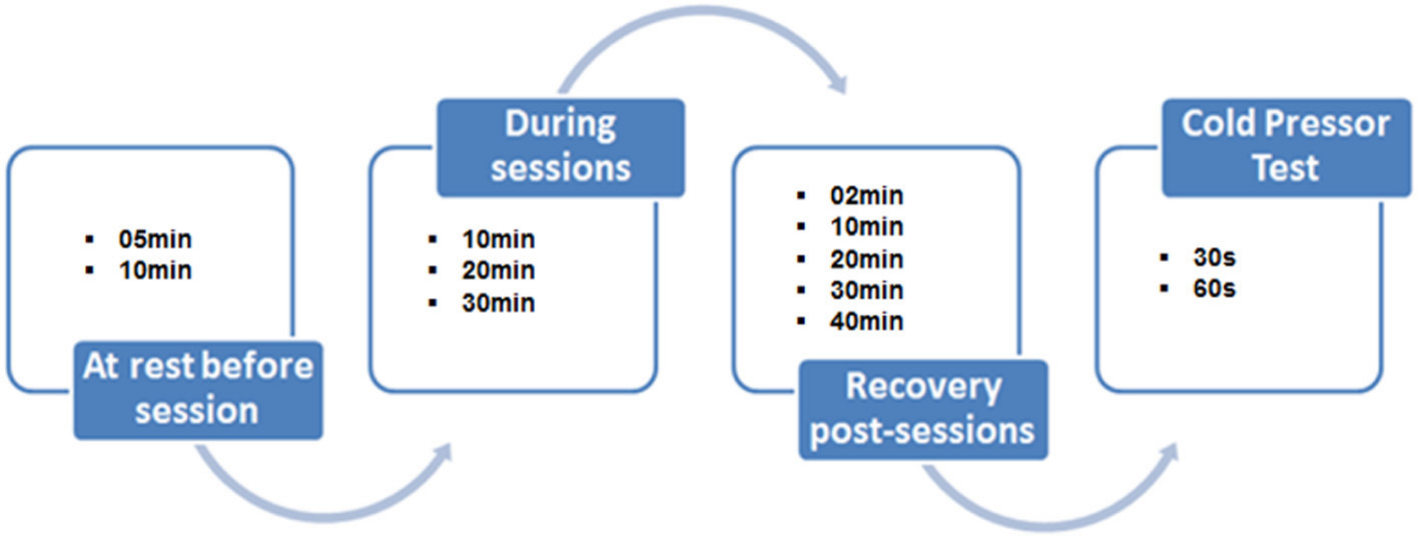
**Study design representing BP and HR measurements during sessions and when performing the Cold Pressor Test**.

Systolic (SBP), diastolic (DBP) BP and HR were collected during each session at the 5th and 10th min of rest before sessions; at the 10th, 20th, and 30th min during sessions; and at the 2nd, 10th, 20th, 30th, and 40th min after all sessions (see Figure 1). Mean arterial pressure (MAP) was calculated using the following equation: MAP = DBP + [(SBP − DBP)/3]. BP was measured, in the right arm, with the volunteer seated, through the auscultatory method using a pediatric sphygmomanometer with an appropriate cuff for children (Becton Dickinson, Brazil) and a children's stethoscope Duo Sonic (Missouri, Brazil). Heart rate was measured using a HR monitor (FS1, Polar Electro Oy, Finland).

#### Cold Pressor Test (CPT)

This thermal stress test was performed after the completion of 40 min of recovery after each session. The volunteers immersed the left hand of the child in cold water (~4 - 5°C) for 60 s. During this period, BP was measured at the 30th and 60th s, and subsequently compared with BP at rest. This test was performed in order to verify the BP response after the different sessions (PLAY, DDR, and TV). BP was measured using auscultatory method by the same evaluator, on the arm opposite to that immersed one, and HR was also measured using the same procedures and equipment cited above.

### Statistical analysis

Shapiro-Wilk's test was used to confirm the normality of data. One-Way ANOVA with Scheffé as a *post hoc* were applied to compare variables between sessions (PLAY, DDR, and TV). ANCOVA for repeated measures adjusted for exercise intensity (i.e., VO_2_) was applied at different time points during recovery and CPT after TV, DDR, and PLAY sessions. The level of significance was set at *p* ≤ 0.05. The data was analyzed using the Statistical Package for the Social Sciences (SPPS), version 15.0 for windows.

## Results

The characteristics of the children were: mean age 9.6 ± 0.5 years, 133.8 ± 9.9 cm of height, weight 32.4 ± 4.0 kg with a mean body mass index (BMI) of 18.4 ± 3.7 kg· m^−2^, and body fat of 17.3 ± 3.8 %. All children were characterized as eutrophic.

Table 1 shows the cardiovascular and ventilatory responses at rest and during each experimental session. Heart rate, SBP, DBP, and MAP were significantly higher during PLAY and DDR when compared to TV. Also, PLAY elicited a significantly higher VO_2_ and RER when compared to TV. Furthermore, all cardiovascular and ventilatory responses were significantly higher during PLAY in comparison to DDR session.

**Table 1:** **Cardiovascular and ventilatory responses at rest and during each session**.

**Variables**	**TV**		**DDR**		**PLAY**	
	**Rest**	**During**	**Rest**	**During**	**Rest**	**During**
HR (bpm)	82.9 ± 5.1	84.9 ± 4.7	85.4 ± 5.4	109.1 ± 9.3^*^	82.6 ± 5.2	144.9 ± 11.3^*^^‡^
SBP (mmHg)	99.6 ± 6.9	99.8 ± 6.2	99.9 ± 6.0	115.5 ± 5.2^*^	99.8 ± 5.8	138.2 ± 7.3^*^^‡^
DBP (mmHg)	63.6 ± 5.0	63.8 ± 5.9	63 ± 5.4	72.6 ± 5.0^*^	63.4 ± 4.7	79.1 ± 3.0^*^^‡^
MAP (mmHg)	75.6 ± 4.6	75.8 ± 5.0	75.3 ± 4.9	86.9 ± 4.1^*^	75.5 ± 4.2	98.8 ± 3.7^*^^‡^
VO_2_ (ml·kg^−1^·min^−1^)	7.1 ± 1.1	7.5 ± 1.0	7.2 ± 1.4	11.7 ± 1.3	7.1 ± 1.5	23.1 ± 4.0^*^^‡^
RER	0.77 ± 0.04	0.76 ± 0.03	0.78 ± 0.04	0.84 ± 0.04	0.76 ± 0.05	0.95 ± 0.08^*^^‡^

Values expressed as mean ± standard deviation. TV, television; DDR, dance dance revolution; PLAY, traditional children's game; HR, heart rate; SBP, systolic blood pressure; DBP, diastolic blood pressure; MAP, mean arterial pressure; VO_2_, oxygen uptake; RER, respiratory exchange ratio.

* p ≤ 0.01 in relation to TV;

‡ *p ≤ 0.01 in relation to DDR*.

**Figure 2:**
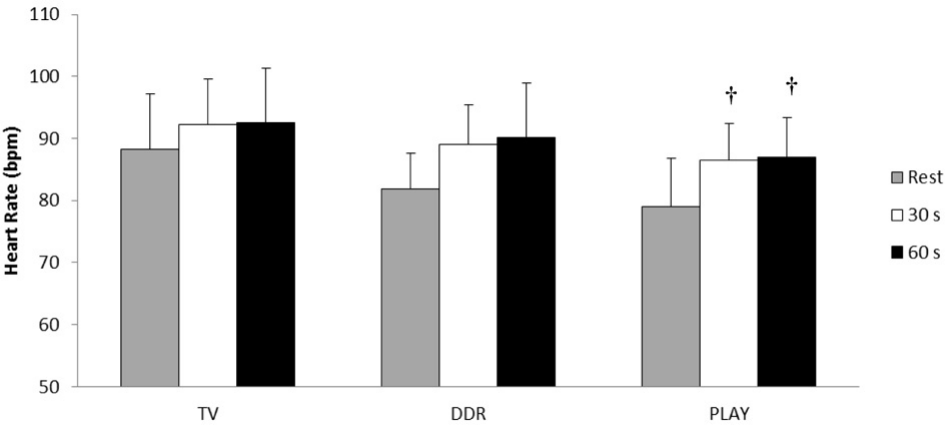
**Heart rate responses at the 30th and 60th s during Cold Pressor Test in each session**. ^†^*p* ≤ 0.05 in relation to pre-exercise rest.

**Figure 3:**
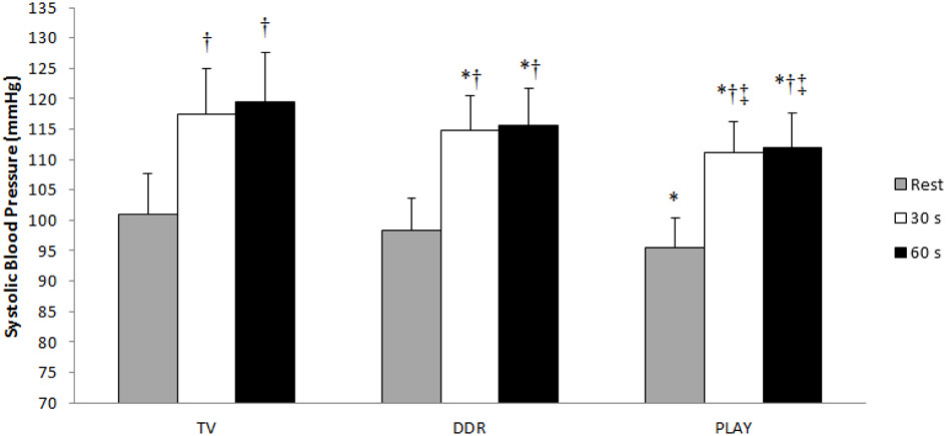
**Systolic blood pressure values during the Cold Pressor Test**. TV, television; DDR, dance dance revolution; PLAY, traditional games; ^*^p ≤0.05 to TV in the same moment; ^†^*p* ≤ 0.05 to rest in the same session; ^‡^*p* ≤ 0.05 to DDR in the same moment.

**Figure 4:**
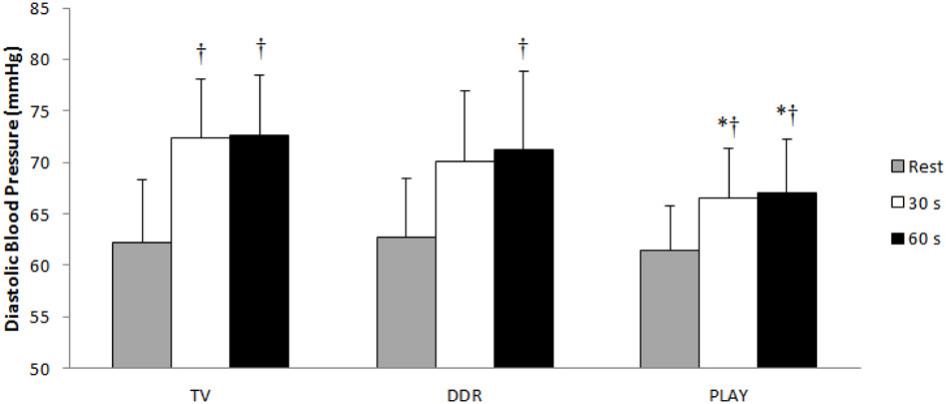
**Diastolic blood pressure values during the Cold Pressor Test**. TV, television; DDR, dance dance revolution; PLAY, traditional games; ^*^p ≤ 0.05 vs. TV in the same moment; ^†^*p* ≤ 0.05 vs. rest in the same session.

Figures 2–**4** illustrate HR and BP responses during CPT. One of the volunteers was considered hyper-reactive to the test, having an increase in SBP of ≥25 mmHg during the test (Wood et al., 1984). Heart rate increased 4, 7.2, and 7.5 bpm at the 30th s, and 4.4, 8.2, and 8 bpm at the 60th s during the CPT after TV, DDR and PLAY, respectively. However, when adjusted for exercise intensity, only PLAY elicited a significant HR increase at both moments when compared to pre-exercise rest.

Figures 3, 4 show the responses of SBP and DBP, respectively, at the 30th and 60th s during CPT after each session. PLAY elicited a significantly smaller increase in SBP and DBP when compared to TV and in SBP when compared to DDR at both moments. When adjusted for exercise intensity, all sessions elicited a significant SBP increase at both moments when compared to pre-exercise rest with the extent of this change in SBP being greater in TV with respect to DDR and PLAY, and lower in PLAY with respect to TV and DDR. Meanwhile, only PLAY exhibited a lower DBP at both moments when compared to TV. Additionally, SBP at rest was significantly lower in PLAY when compared to TV.

Tables 2, 3 show the mean values of SBP and DBP over each session and subsequent recovery. With or without adjustment for exercise intensity, SBP and DBP in DDR and PLAY were significantly higher at the 10th, 20th, and 30th min of intervention when compared to TV. PLAY also showed significantly higher values when compared to DDR at all times for SBP and DBP, but only at the 10th and 20th for DBP when controlled for exercise intensity. During the recovery period, SBP and DBP were significantly reduced after adjustment for exercise intensity at the 30th min, and SBP at the 40th min when compared to rest only in PLAY. Interestingly, SBP at 2nd min of recovery in DDR was different from rest in the same session before adjustment for exercise intensity. Moreover, SBP at the 40th min of recovery and DBP at the 2nd of recovery in PLAY were significantly different from the values recorded after TV session before adjustment for exercise intensity. In contrast, DBP was not different from values recorded after TV at the 30th and 40th min of recovery before adjustment for exercise intensity.

**Table 2:** **Systolic blood pressure mean values (±*SD*) of all sessions (TV, DDR, and PLAY) (*n* = 18)**.

	**Moment**		
	**TV (mmHg)**	**DDR (mmHg)**	**PLAY (mmHg)**
**PRE-INTERVENTION REST**			
	100.4 ± 7.1	101.6 ± 6.5	100.1 ± 5.8
**SESSIONS**			
10 min	101.1 ± 6.2	114.8 ± 4.9^*^^†^	137.9 ± 7.7^*^^†^^‡^
20 min	99.7 ± 6.5	116.3 ± 5.0^*^^†^	139.4 ± 6.6^*^^†^^‡^
30 min	100.4 ± 6.4	116.4 ± 5.1^*^^†^	138.2 ± 6.8^*^^†^^‡^
**POST-INTERVENTION RECOVERY**			
2 min	99.8 ± 6.6	105.6 ± 4.8^*^	109.1 ± 6.0^*^^†^
10 min	100.9 ± 6.7	101.4 ± 4.8^§^	101.5 ± 5.0^§^
20 min	100.7 ± 6.4	100.2 ± 5.1^§^	99.0 ± 5.2^§^^#^
30 min	101.8 ± 7.0	99.3 ± 5.6^§^	97.1 ± 5.7^†^^§^^#^
40 min	101.3 ± 6.8	98.8 ± 5.1^§^	95.8 ± 4.7^†^^§^^#^

TV, television; DDR, dance dance revolution; PLAY, traditional games;

* p ≤ 0.05 vs. TV in the same moment;

† p ≤ 0.05 vs. rest in the same session;

‡ p ≤ 0.05 vs. DDR in the same moment;

§ p ≤ 0.05 vs. 2 min recovery in the same session;

# *p ≤ 0.05 vs. 10 min recovery in the same session*.

**Table 3:** **Diastolic blood pressure mean values (±*SD*) of all sessions (TV, DDR, and PLAY) (*n* = 18)**.

	**Moment**		
	**TV (mmHg)**	**DDR (mmHg)**	**PLAY (mmHg)**
**PRE-INTERVENTION REST**			
	64.5 ± 4.8	63.8 ± 5.3	64.1 ± 4.8
**SESSIONS**			
10 min	64.9 ± 5.8	71.9 ± 4.8^*^^†^	78.7 ± 3.7^*^^†^^‡^
20 min	64.7 ± 6.4	73.4 ± 4.7^*^^†^	80.2 ± 3.1^*^^†^^‡^
30 min	65.2 ± 6.4	73.9 ± 5.5^*^^†^	79.5 ± 2.3^*^^†^
**RECOVERY**			
2 min	64.5 ± 6.2	68.7 ± 5.9^†^	69.6 ± 4.2^†^
10 min	65.5 ± 5.7	66.0 ± 6.4^§^	65.2 ± 4.4^§^
20 min	65.8 ± 5.6	64.6 ± 6.0^§^	64.0 ± 4.8^§^
30 min	65.8 ± 5.7	64.2 ± 6.3^§^	62.7 ± 4.8^*^^§^
40 min	66.1 ± 6.0	63.9 ± 5.9^§^	62.1 ± 4.6^*^^†^§^^

TV, television; DDR, dance dance revolution; PLAY, traditional games;

* p ≤ 0.05 vs. TV in the same moment;

† p ≤ 0.05 vs. rest in the same session;

‡ p ≤ 0.05 vs. DDR in the same moment;

§ *p ≤ 0.05 in relation to 2 min recovery in the same session*.

## Discussion

The main findings of present study were that children experienced PEH after traditional games and that BP reactivity to stress induced by CPT was also lower after traditional games when compared to active video game playing and TV sessions. These responses may be related to exercise intensity once the more intense session (PLAY) also induced a more significant hypotensive effect during the post-exercise recovery period. Moreover, this lowered BP reactivity was observed 40 min after the end of the experimental session, which is longer than that observed in previous studies (Roemmich et al., 2009; Lambiase et al., 2010). Overall, these findings suggest that traditional games are better suited for cardiovascular health in children when compared to other activities of lower cardiovascular and metabolic stimuli like active video game playing or watching TV.

To the best of our knowledge, this is the first study reporting PEH in children. Thus, significant reductions in SBP and DBP were observed in children at the 30th and 40th min of recovery when compared to rest only after PLAY. Interestingly, DBP was significantly reduced when compared to TV session after adjustment for exercise intensity. In contrast, SBP at the 40th min of recovery was not different from SBP values recorded after TV session when controlled for exercise intensity. From a methodological point of view, these findings are interesting and suggest the necessity in further studies of comparing the effect of different activities matched for exercise intensity on cardiovascular responses. Additionally, as the recovery period lasted only 40 min in the current study, further studies should evaluate if the PEH exerts its positive influence over longer periods in children.

Previously, Roemmich et al. (2009) reported that previous interval exercise protocol in cycloergometer also dampened the subsequent cardiovascular response to a speech stressor test when compared to watching TV. Similarly, Lambiase et al. (2010) have reported that the cardiovascular reactivity during cognitive stress was dampened after a treadmill walking when compared to a simulated sedentary drive to school. These results are consistent with the results of the current study, and also with previous studies in adults (Probst et al., 1997; Bond et al., 2002), thus confirming the positive impact that acute exercise has on stress tolerance. However, this is the first study that reproduces traditional games which is a natural condition that imitates the habitual patterns of PA in children. Furthermore, the greater positive impact of traditional games (PLAY) could be due to its higher intensity as inferred from the higher exercising HR, BP, and VO_2_ recorded when compared to active video game playing, which have exhibited similar values as in previous reports (Wang and Perry, 2006). Therefore, this suggests that street playing in a non-controlled environment is an effective way for reducing the cardiovascular reactivity in children. PLAY seems to be more motivating, thus naturally increasing the levels of PA in an easy manner.

While we did not evaluate the underlying mechanisms that accounts for these differences, it may be suggested that the greater intensity achieved by children during street playing, as reflected in a greater HR increase (i.e., 72% for HR_max_ during PLAY vs. 54% HR_max_ during DDR; Machado and Denadai, 2011), and thus the occurrence of PEH after this more intense intervention, may have a role in a lowered post-exercise BP reactivity. Previous studies have reported that a greater sympathetic activation during exercise could mediate a lower post-exercise reactivity to stress (Lovallo, 2005). In this regard, Brownley et al. (2003) have demonstrated that a single bout of aerobic cycle exercise in adults reduces the post-exercise norepinephrine production in response to stress, favoring a lower cardiovascular reactivity that could also be related to a greater vasodilatory effect of exercise. Additionally, Negrão et al. (2003) have also suggested that exercise could induce a greater nitric oxide release after exercise that could elicit the vasodilatation response, and consequently favor both the PEH and the lower BP reactivity to post-exercise stress. As we did not evaluate possible mechanisms in the current study, it may be suggested that exercise intensity and subsequent metabolic demands could be the major factor accounting to these differences between conditions, as recently reported (Eicher et al., 2010; Simões et al., 2010). Additionally, the significant increase in HR during the CPT after PLAY that was only evident when cardiovascular responses were controlled for exercise intensity, may suggest the existence of other mechanisms (e.g., nitric oxide) different from autonomic control that may account for both the reduced BP reactivity and PEH as suggested in a previous study with women exhibiting PEH after resistance exercises (Tibana et al., 2013). Therefore, further studies are needed in order to determine the exact physiological mechanisms that account to these differences in cardiovascular reactivity in children after exercise, considering exercise mode and intensity.

The current study has some limitations. First, the number of boys and girls is limited and do not allow comparisons between genders. Second, the CPT does not reproduce the habitual source of stress in children's daily activities although is a valid, simple and time saving tool for cardiovascular stress reactivity evaluation. Therefore, further studies should be conducted for testing the potential differences between boys and girls regarding other stressors, such as those experienced at school, in a more realistic environment. Third, we only recorded cardiovascular responses within 40 min of recovery. Therefore, it is unknown how these responses could be maintained in the long-term (e.g., 24 h). Lastly, it is well known that genetic factors and ethnicity could be related to hypertension in adulthood (Douglas et al., 2003), but no evidence is available regarding such differences in children. In this scenario, as our sample was composed of blacks and whites, it may be interesting to look for such potentially genetic differences in further studies.

## Conclusion

The most relevant finding of the present study was that the 30-min exercise session based on traditional games (“run and catch,” “dodge ball,” and “capture the flag”) resulted both in PEH and lower BP reactivity to stress in children. These results could be due to greater exercise intensity and metabolic demands recorded during traditional games. Additional studies should be conduct to elucidate the influence of intensity, mode and duration of activities usually performed by children, the impact of these variables, and the possible mechanisms underlying the protective cardiovascular effect during subsequent stress-induced BP reactivity.

### Conflict of interest statement

The authors declare that the research was conducted in the absence of any commercial or financial relationships that could be construed as a potential conflict of interest.
